# Orthopedic manifestations in children with Prader-Willi syndrome

**DOI:** 10.1186/s12887-024-04603-7

**Published:** 2024-02-14

**Authors:** Miao Miao, Guo-Qiang Zhao, Qiong Zhou, Yun-Qi Chao, Chao-Chun Zou

**Affiliations:** 1 Department of Endocrinology, The Children’s Hospital of Zhejiang University School of Medicine, No. 3333, Binsheng Road, Hangzhou, 310052 China; 2 Department of Emergency Trauma, The Children’s Hospital of Zhejiang University School of Medicine, Hangzhou, 310052 China; 3 Hangzhou Children’s Hospital, No. 195 Wenhui Road, Hangzhou, 310000 China

**Keywords:** Prader-Willi syndrome, Scoliosis, Hip dysplasia

## Abstract

**Background:**

Prader-Willi syndrome (PWS) is a rare genetic disease often associated with bone problems, mainly scoliosis and hip dysplasia (HD). This study aimed to analyze the clinical characteristics of orthopedic deformities in patients with PWS.

**Methods:**

A retrospective study was conducted on 175 patients up to March 2023. The Cobb angle(CA) of the spine, the alpha angle of the hip joint, and the acetabular index (AI) were measured. This study aimed to evaluate the relationship between demographic parameters and bone deformities.

**Results:**

Scoliosis was found in 66 patients (43.7%), including 52 (78.8%) with mild scoliosis, 10 (15.2%) with moderate scoliosis, and 4 (6.1%) with severe scoliosis. Only seven patients received orthopedic treatment (10.6%). The median age of scoliosis was 4.5 years old, and the prevalence of scoliosis increased rapidly at the age of 5 years and adolescence. The mean CA in this study increased gradually with age. HD was found in 47 patients (38.2%), and 6 patients received orthopedic treatment (12.7%). The median age at HD was 1.8 years old. The mean AI of the study population decreased with age. The prevalence of HD treated with recombinant human growth hormone (rhGH) was low. No significant differences were observed in sex, genotype, body mass index (BMI), obesity rate, or onset of scoliosis and HD.

**Conclusion:**

The prevalence of scoliosis and HD was higher in patients with PWS. The onset age and developmental trends of the different skeletal malformations were different. Early diagnosis and treatment are important for the prognosis and treatment of orthopedic diseases in patients with PWS.

## Introduction

Prader-Willi syndrome (PWS) is a multisystem hereditary disease with an incidence of 1/10 000–1/30 000. This results from the lack of expression of paternal genes in the 15q11.2-q13.1 region. There are 3 main types of PWS, including paternal deletion (65-75%), maternal uniparental disomy (mUPD) (20 -30%), and imprinting defects (1-3%).^1^ PWS displays significant clinical variability with age, ranging from hypotonia and sucking weakness during infancy to hyperphagia, morbid obesity, hypogonadism, and growth retardation [1–4]. 

PWS is commonly associated with orthopedic deformities, including scoliosis, hip dysplasia (HD), foot deformities, ligament relaxation, and osteoporosis [5]. The incidence of skeletal deformities is higher in PWS. Scoliosis is present in 30-78% of patients [6, 7]. Scoliosis may affect the motor function of respiratory muscles and lead to respiratory damage [8]. Cor pulmonale and respiratory failure might be the most common cause of death and long-term complications in PWS [7, 9, 10]. Recombinant human growth hormone (rhGH) is one of the drugs used in PWS, but its effect on bone deformity is still controversial. rhGH therapy has been considered a possible risk factor for the onset of scoliosis in some studies [11]. However, others have demonstrated that rhGH therapy is not related to the occurrence and development of scoliosis [7, 12, 13]. This may be related to the population, dose, and age of the patient. However, large-sample studies in patients with scoliosis treated with rhGH are still lacking. At present, rhGH is used at a younger age [14]. We should pay more attention to its effects on bones such as the spine. The prevalence of HD in PWS is higher than that in the non-PWS population, accounting for 10–48% [1, 15–19]. Early treatment with rhGH may improve the progression of HD [20]. Approximately 30% of patients with PWS with HD need to receive orthopedic treatment [15]. It may develop osteoarthritis of the hip joint in the later stage of HD [21]. Therefore, early orthopedic intervention for HD is necessary.

This study summarized the clinical characteristics of skeletal deformities in Chinese patients with PWS and analyzed the risk factors for orthopedic malformation, especially focusing on sex, genotype, rhGH therapy, and body mass index (BMI).

## Materials and methods

### Subjects

Patients registered at the Children’s Rare Disease Care Center and PWS Rare Disease Care Center before March 2023 were recruited for this study. All the patients were genetically confirmed. The clinical and radiographic data were reviewed.

The study protocol was approved by the Ethical Committee of the Children’s Hospital of Zhejiang University School of Medicine. Informed consent was obtained from all parents registered in the PWS Registry.

A team of professional pediatric orthopedic surgeons evaluated and diagnosed all patients. Scoliosis was diagnosed as a Cobb angle (CA) > 10° and divided into 3 degrees according to the Scoliosis Research Society classification [22]: mild scoliosis as CA between 10° and 20°, moderate scoliosis as CA between 20° and 40°, and severe scoliosis as CA > 40°. The range of scoliosis was evaluated according to the Lenke scoliosis [23]. 

The diagnostic criteria for HD differ according to age. In patients aged < 6 months, an ultrasonic α angle of the hip joint < 60° and femoral head coverage < 50% were diagnosed with HD. In patients aged ≥ 6 months, hip joint development was evaluated using the acetabular index (AI). HP was defined as AI > 30° at an age between 6 months and 1 year, and AI > 25° at an age of over 1 year [24]. 

All patients underwent height/length and weight measurements while wearing a light underwear. The body weight of patients under 2 years of age was obese when the WHO weight Z value was > 3 [25] The percentage of BMI in the body mass index growth curve of patients over 2 years of age was more than 95% [26]. 

Statistical analyses were performed using SPSS (version 25.0). Descriptive statistics were used to summarize the demographic and clinical characteristics of the patients. Measurement data are presented as median (min–max). The differences in age, BMI, and CA between the groups were analyzed using the nonparametric Kruskal–‒Wallis test. Enumeration data are presented as frequencies and percentages. Differences between the groups were compared using the chi-square test. Logistic regression analysis was used to analyze the scoliosis and HD risk factors. Statistical significance was set at *p* < 0.05.

## Result

### Characteristics of the cohort

A total of 175 patients, including 89 men (50.9%) and 86 women (49.1%), were enrolled. The median age of diagnosis was 0.3 years (0.1–3.7 years). A total of 107 (61.1%) patients had a deletion genotype, 45 (25.7%) had a non-deletion type, including mUPD and imprinting defects, and 23 (13.1%) were diagnosed with methylation-sensitive polymerase chain reaction (MS-PCR), but the genotype was not clear. A total of 149 (85.1%) patients received rhGH therapy. A total of 151 patients (86.3%) underwent spinal radiography and 122 (69.7%) underwent hip X-ray/hip ultrasound evaluation.

### Scoliosis and risk factors

**Fig. 1:**
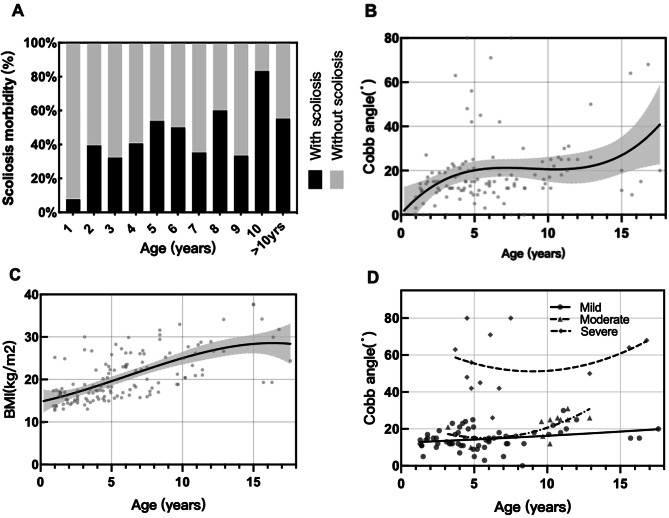
Clinical data analysis of scoliosis. (**A**) Prevalence of scoliosis in different age groups. (**B**) Regression lines of age fitted for the CA. (**C**) Curve of BMI and age changes in patients with scoliosis (**D**) Changes in CA with age in different severity groups

Among the 151 PWS patients, 66 (43.7%) developed scoliosis, with a median age of 4.5 years. The prevalence of scoliosis increased rapidly at the age of 5 and adolescence (Fig. 1-A). The degree of scoliosis increased with age and worsened before 5 years and approximately 15 years of age (β coefficient, 7.54; 95% CI, 1.59–13.49) (Fig. 1-B). The BMI in PWS between patients with scoliosis and those without scoliosis increased before the age of 15 years, and there was no difference between the two groups (*p* = 0.821) (Fig. 1-C). In addition, there were no significant difference**s** between sex, genotype, rhGH onset, obesity rate, and the risk of scoliosis (Table 1).

**Table 1:** Analysis of risk factors for scoliosis in PWS patients

	Scoliosis	Nonscoliosis	OR (95%CI)	*p* value
**Number**	66 (43.7%)	85 (56.3%)		
**Gender (M/F)**	32/34	44/41	0.74(0.38–1.47)	0.393
**Genotype**				
Deletion	41 (45.6%)	49 (54.4%)	-	-
Nondeletion	20 (50.0%)	20 (50.0%)	1.31(0.60–2.86)	0.492
Other	5 (23.8%)	16 (76.2%)	0.39(0.13–1.18)	0.097
**rhGH Therapy (yes/no)**	54/12	62/23	1.77(0.72–4.31)	0.213
**rhGH therapy onset age (yrs)**	1.2(0.1–15.1)	1(0.1–11.7)	1.05(0.94–1.18)	0.357
**BMI (kg/m** ^**2**^ **)**	17.9(13.0-37.7)	18.1(10.9–24.3)	1.04(0.94–1.14)	0.438
**Obesity (yes/no)**	29/37	39/46	0.79(0.26–2.39)	0.674

Abbreviations: M male, F femal, yrs years

The results are given as median

### Severity of scoliosis

In the cross-sectional data analysis, scoliosis was mild in 52 patients (78.8%), moderate in 10 (15.2%), and severe in 4 (6.1%). There were more patients with scoliosis bending to the right (66.7%), but there was no difference in the direction of scoliosis with different severities (*p* = 0.624). The proportions of thoracic and lumbar vertebrae as the primary sites involved were similar (39.4% and 47.1%, respectively). Moderate and severe scoliosis were more prevalent in the lumbar spine, shown as type 5 and type 6 in the Lenke classification. (*p* = 0.006). There was no difference in sex, genotype, rhGH onset, obesity rate, or age at PWS diagnosis (Table 2).

**Table 2:** Characteristics of 66 PWS patients stratified by severity of scoliosis

	Overall	Mild	Moderate	Severe	χ^2^ / Z	*p* value
**Gender (M/F)**	32/34	25/27	5/5	2/2	0.19	1.000
**PWS onset age (yrs)**	0.9(0.1–16.4)	0.7(0.1–16.3)	0.9(0.1–12.2)	4.4(0.6–15.6)	2.34	0.311
**Genotype**						
Deletion	41(62.1%)	34(82.9%)	4(9.8%)	3(7.3%)	7.28	0.082
Nondeletion	20(30.3%)	16(80.0%)	4(20.0%)	0		
Others	5(7.6%)	2(40.0%)	2(40.0%)	1(20.0%)		
**rhGH therapy (yes/no)**	54/12	43/9	8/2	3/1	0.70	0.855
**rhGH therapy onset age (yrs)**	1.1 (0.1–15.1)	1.2 (0.1–15.1)	2.6 (0.8–12.3)	0.8(0.2–6.4)	2.98	0.226
**BMI (kg/m** ^**2**^ **)**	18.1(10.9–37.7)	18.0(13.0-37.7)	17.9 (14.5–26.7)	17.2(15.0-34.2)	0.02	0.992
**Obesity (yes/no)**	29/37	24/28	3/7	2/2	1.03	0.650
**Side of major curve (R/L)**	44/22	36/16	6/4	2/2	1.15	0.624
**Lenke classification**						
Type 1	25(37.9%)	22(88.0%)	2(8.0%)	1(4.0%)	17.91	**0.006**
Type 2	1(1.5%)	1(100.0%)	0	0		
Type 3	9(13.6%)	6(66.7%)	2(22.2%)	1(11.1%)		
Type 5	25(37.9%)	22(88%)	3(12.0%)	0		
Type 6	6(9.1%)	1(16.7%)	3(50.0%)	2(33.3%)		

Abbreviations: M male, F femal, R right, L left, yrs years

*p* value less than 0.05 is expressed in bold

When we tracked the change in CA according to different severities, as shown in Fig. 1-D, the angle of the moderate and severe groups tended to increase with age, while the angle of the mild group changed slowly.

### Orthopedic treatment of scoliosis

**Fig. 2:**
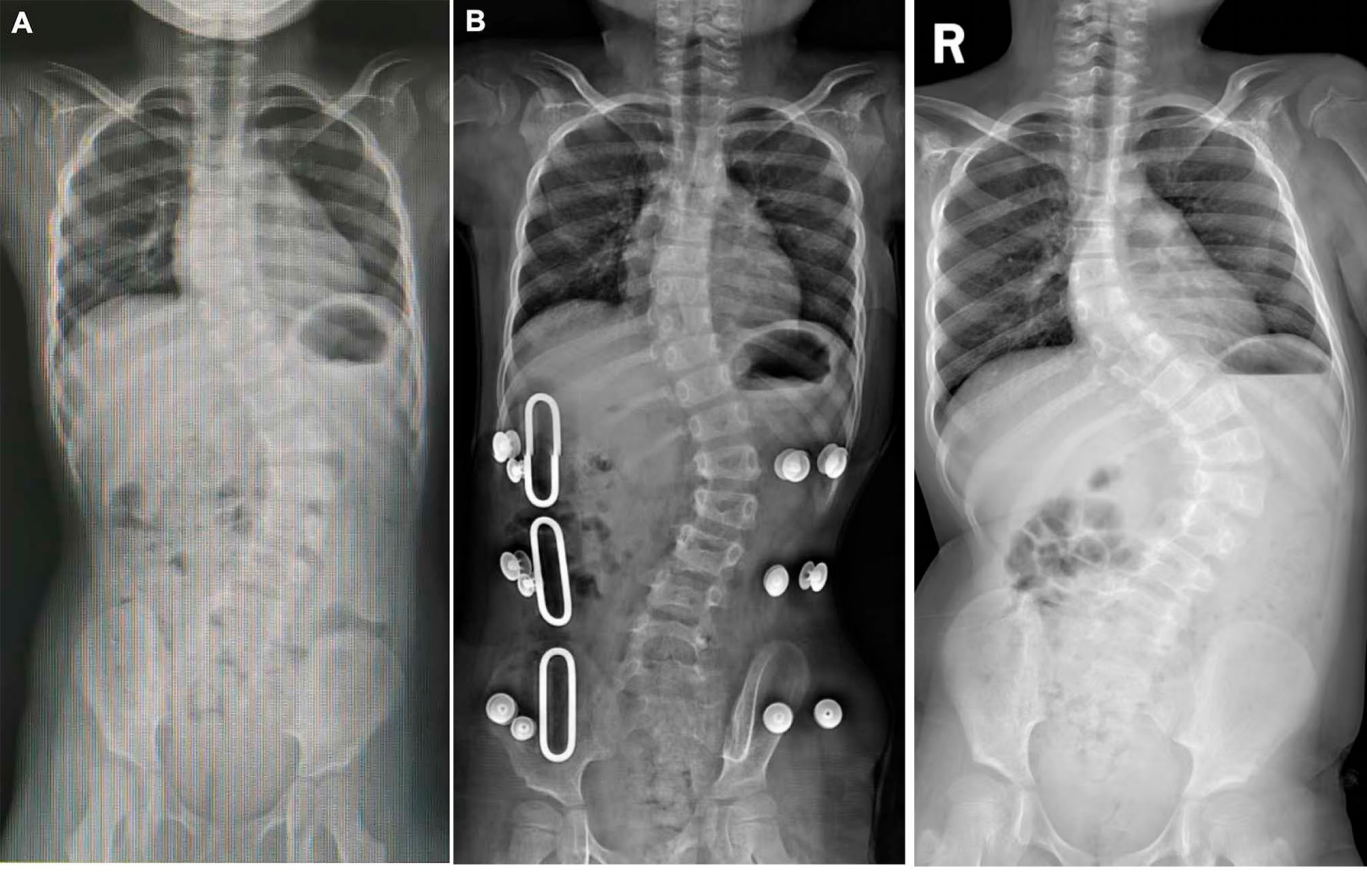
Changes in scoliosis in patient 3 before and after brace treatment. (**A**) Before brace treatment, 4.5 years old, thoracic curvature 29 degrees, lumbar curvature 48 degrees. (**B**) After 1.5 years of brace treatment, the thoracic curvature was 26 degrees, and the lumbar curvature was 26 degrees. (**C**) After removing the brace fixation, the 7.5-year-old thoracic vertebra arc was 48 degrees, and the lumbar vertebra arc was 80 degrees

Seven patients (10.6%) received orthopedic treatment, surgical treatment in 1 patient, and 6 patients received brace treatment. Before treatment, four patients had moderate scoliosis, and three patients had severe scoliosis. Two patients who were followed up after treatment were lost to follow-up. The average follow-up time was 29.8 months, of which the average duration of brace treatment was 18.3 months (range: 6–34 months), and the treatment time of patients with internal fixation was 6.7 years. Scoliosis was aggravated in patients 1 and 2 after treatment, and improved in the other patients (Table 3). Patient 3 had recurrence of scoliosis after stopping brace treatment (Fig. 2).

**Table 3:** Follow-up of patients with scoliosis undergoing orthopedic treatment

Patient	Scoliosis onset age(yrs)	Gender	Weight	CA before treatment(°)	Outcome (°)	Treatment	Follow-up (m)
1	9.6	M	Overweight	25°	31°	Spinal Brace	6
2	10.9	M	Overweight	25°	33°	Spinal Brace	34
3	4.5	F	Normal	56°	26°	Spinal Brace	15
4	4.5	M	Obesity	71°	50°	Internal Fixation	80
5	3.7	F	Obesity	63°	42°	Spinal Brace	14

Abbreviations: M male, F femal, CA cobb angle, yrs years,m month.

### HD and risk factors

**Fig. 3:**
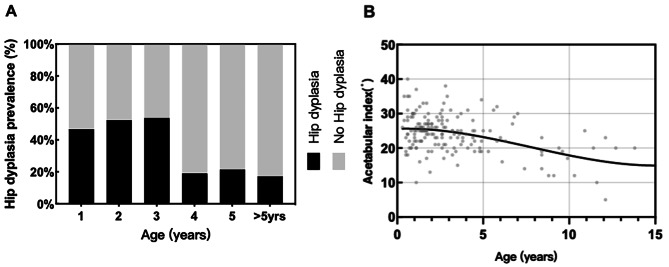
Correlation of AI and the prevalence of HD with age. (**A**) Relationship between prevalence rate and age of HD. (**B**) The trend of the AI with age

Of the 123 patients who participated in hip development assessment, 47 (38.2%) had HD, of which 3 (6.4%) had hip dislocation.The median age of HD onset was 1.7 years (range 0.3 to 6.8 years), mainly in patients younger than three years (Fig. 3-A). The prevalence of HD with rhGH therapy was lower(*p* = 0.036). AI decreased during patient growth, as shown in (Fig. 3-B). There were no differences in sex, genotype, BMI, obesity rate, or scoliosis complications in the risk of HD (Table 4).

**Table 4:** Analysis of risk factors for HD in PWS patients

	HD	Non-HD	OR (95% CI)	*p* value
**Number**	47(38.2%)	76(61.8%)		
**Gender (M/F)**	22/25	42/34	1.16(0.48–2.85)	0.741
**Genotype**				
Deletion	25(33.3%)	50(66.7%)	-	-
Nondeletion	19(52.8%)	17(47.2%)	1.20(0.46–3.15)	0.715
Other	3(25.0%)	9(75.0%)	0.64(0.14–1.87)	0.563
**rhGH therapy (yes/no)**	38/9	63/13	0.07(0.01–0.84)	**0.036**
**rhGH therapy onset age (yrs)**	0.7(0.1–6.7)	1.2(0.1–11.7)	0.68(0.46–1.03)	0.065
**BMI (kg/m2)**	15.6(11.0-27.1)	17.3(12.0-34.1)	0.86(0.72–1.03)	0.109
**Obesity (yes/no)**	4/43	22/54	1.05(0.09–12.4)	0.970
**With Scoliosis (yes/no)**	13/33	30/46	0.56 (0.22–1.40)	0.216

Abbreviations: M male, F femal, HD hip dysplasia, yrs years

*p* value less than 0.05 is expressed in bold

### Orthopedic treatment of HD

**Fig. 4:**
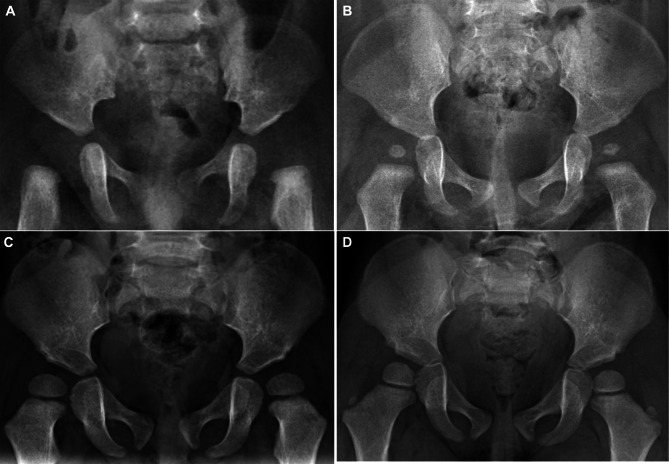
Patients with hip dysplasia that resolved after treatment. (**A**) Patient 2 before surgery. (**B**) Patient 2 after surgery. (**C**) Patient 5 before Pavlik hardness treatment. (**D**) Patient 5 after Pavlik hardness treatment

Of the 47 patients, six (12.7%) received orthopedic treatment, of which four received Pavlik (mean age at diagnosis: 1.2 years) and two received surgical treatment (mean age at diagnosis: 0.8 years). The mean follow-up was 9.5 months. HD resolved in patients 2 and 5 (Fig. 4), and improved in the other five patients (Table 5).

**Table 5:** Patients with HD participating in orthopedic treatment

Patient	HP onset age (yrs)	Gender	AI before treatment		Outcome		Treatment	Follow-up
			R	L	R	L		(m)
1	1.1	M	32	37	27	26	Surgery	8
2	0.4	F	35	35	24	23	Surgery	7
3	0.6	F	35	35	25	28	Pavlik harness	14
4	0.9	F	29	30	24	26	Pavlik harness	6
5	1.0	F	27	27	21	21	Pavlik harness	16
6	2.3	F	29	26	26	25	Pavlik harness	6

Abbreviations: M male, F femal, R right, L left, HD hip dysplasia yrs years, m month

## Discussion

In this study, the incidence of scoliosis was 43.7%, similar to previous studies [27] and much higher than the 1–3% incidence of scoliosis in the average population [28]. The median age of onset was 4.5 years old, which is lower than the French(12.9 years) and Italian(6.3 years) data [7, 16]. In our study, 86.3% of PWS patients underwent spinal radiography, which may be related to early monitoring of scoliosis, indicating an increase in awareness of PWS scoliosis.

In the present study, the degree of scoliosis increased with age, similar to that observed in idiopathic scoliosis [29]. The trend of CA increase with age is more evident at the ages of 5 and 15 years, similar to the two peaks of the incidence of scoliosis at the age of 5 years and puberty. This suggests a rapid growth rate and curve progression caused by increased spinal growth rate. There were no significant differences in the incidence of scoliosis among the different genotypes in patients with PWS. Genetic differences exist in the incidence of scoliosis in adult patients with PWS, and the deletion type is more common [30]. This may be related to the fact that the spine is still in the developmental stage in childhood, and scoliosis may continue to progress. Sex is not a risk factor for scoliosis in patients with PWS, but the incidence is higher in women with idiopathic scoliosis [29]. rhGH therapy did not increase the incidence of scoliosis and had no effect on the aggravation of scoliosis in our study. One study showed that rhGH increases the risk of scoliosis in adolescents [11]. A larger sample of data may be needed for support. Current research has shown that the long-term use of rhGH can improve the body composition of patients [14]. Scoliosis should not be considered a reason for refusing rhGH treatment. This study found no association between BIM and CA in PWS patients. This study found that kyphosis is associated with increased BMI [16]. However, there were no patients with kyphosis in our data, so we did not analyze them. Owing to the poor effect of braces in treating scoliosis in obese patients, surgical treatment is often needed. Obese patients have surgical risks, such as respiratory depression [27]. Therefore, weight control progress is essential for patients with scoliosis.

The main direction of the scoliosis was on the right side. Scoliosis occurs in roughly the same proportion of the thoracic and lumbar vertebrae; however, in previous studies, it mainly occurs in the lumbar spine in patients with WPS. This may be related to our finding that the age of onset of scoliosis is earlier and the paraspinal muscle burden of the lumbar spine is lower. According to the different severity groups, mild scoliosis accounted for the majority, and moderate and severe scoliosis mainly occurred in the lumbar vertebrae and thoracolumbar vertebrae, which may be related to the support of ribs in the thoracic vertebrae and the low volume of paraspinal muscles in the L4 plane [31]. The changing trend of scoliosis patients with different severities varies with age. The changes in scoliosis in the mild group tended to be stable, whereas those in the moderate and severe groups worsened with age. This implies that the more obvious the scoliosis is, the more likely it is to progress.

In this study, 10.6% of patients with scoliosis received orthopedic treatment, which was lower than that in previous studies [7, 32]. One of the patients with severe scoliosis had no corrective records, which may be related to the family’s financial burden. One patient with severe scoliosis underwent surgery. The CA of this patient was > 70 °. Patients with mild scoliosis are usually treated with rehabilitative exercise training. Avoiding standing prematurely during childhood to increase the burden on the spine can reduce the risk of scoliosis. Patients with CA > 25 °can be treated with braces, casting, or other orthopedic treatments. For patients with conservative scoliosis whose curve cannot be maintained, the CA is usually greater than 70 °, and surgical treatment is optional [27]. Patient 4 had an aggravated curve after the end of brace treatment, for which surgical treatment may be recommended. The function of brace therapy is to help patients avoid premature surgery.

The incidence of HD was 38.2%, which was at least twice that in the non-PWS population. The median age of onset was 1.8 years old, which is younger than that in a previous study [15]. This finding suggested that there has been an increase in awareness of hip development in patients with PWS in recent years. Dystonia, ligament relaxation, and morbid sleepiness in infants are considered risk factors for HD [7, 15]. There was no correlation between sex and prevalence of HD. Female gender is a risk factor for HD in the non-PWS population, and estrogen is thought to increase ligament relaxation [33]. This may be related to a decrease in gonadal function and estrogen levels in patients with PWS [15]. There was no correlation between the genotype, BMI, and prevalence of HD. In this study, the incidence of HD was lower in the rhGH treatment group, which may be due to the effects of rhGH therapy on muscle growth and weight-bearing capacity.

The proportion of orthopedic treatment in HD patients was 12.7%, which was lower than that in the 2018 study. This may be related to early rehabilitation exercise for HD. The AI of Patient 3 is in the standard high line and still participates in orthopedic treatment, which is probably related to the active treatment requirements of parents. General HD in this study tended to improve with age. Previous research has revealed that HD may be caused by acetabular stunting in these infants, which can be improved with an increase in growth and activity. This may explain why the incidence of HD in adults with PWS is lower than that in children [34]. Studies have shown that PWS in patients who receive treatment for HD may be reversed after complete remission a few years later. The asymptomatic mild residual acetabular dysplasia may also worsen [15]. Thus, it is necessary to conduct a continuous radiological evaluation of the hip joint in these patients and continuously evaluate the level of development of the hip joint.

The limitation of our study is that, first, the prevalence in our study may have been underestimated because we excluded 20 patients without spinal X-ray data and 53 patients without hip joint data. Second, some patients continued to grow and develop at the time of data collection, and skeletal deformities may develop further, requiring a longer follow-up time. Third, the study mainly included data on scoliosis and HD and lacked analysis of other skeletal deformities, such as kyphosis and foot deformities.

In summary, the prevalence of scoliosis and HD is higher in patients with PWS. Therefore, rhGH therapy may reduce the incidence of HP. There are differences in the age of onset of different skeletal deformities, which may be closely related to age. In fact, patients with PWS undergo hip joint evaluation in infancy, and the spine is evaluated later. Patients with PWS should increase the evaluation of orthopedic diseases in the initial stage of the disease, which will benefit the treatment of orthopedic deformities.

## Data Availability

No datasets were generated or analysed during the current study.

## Abbreviations

PWS: Prader Willi syndrome
HD: Hip dysplasia
CA: Cobb angle
AI: Acetabular index
BMI: Body mass index
mUPD: Maternal uniparental disomy
rhGH: Recombinant human growth hormone
MS-PCR: Methylation-sensitive polymerase chain reaction
